# Distinct Types of Feeding Related Neurons in Mouse Hypothalamus

**DOI:** 10.3389/fnbeh.2016.00091

**Published:** 2016-05-18

**Authors:** Yan Tang, Diego Benusiglio, Valery Grinevich, Longnian Lin

**Affiliations:** ^1^Key Laboratory of Brain Functional Genomics (Ministry of Education and Shanghai), School of Life Science and the Collaborative Innovation Center for Brain Science, Institute of Brain Functional Genomics, East China Normal UniversityShanghai, China; ^2^Schaller Research Group on Neuropeptides at German Cancer Research Center, Central Institute of Mental Health, and Cell Networks Cluster of Excellence at the University of HeidelbergHeidelberg, Germany

**Keywords:** hypothalamus, feeding-related neurons, firing patterns, feeding behavior, food intake, *in vivo* recording

## Abstract

The last two decades of research provided evidence for a substantial heterogeneity among feeding-related neurons (FRNs) in the hypothalamus. However, it remains unclear how FRNs differ in their firing patterns during food intake. Here, we investigated the relationship between the activity of neurons in mouse hypothalamus and their feeding behavior. Using tetrode-based *in vivo* recording technique, we identified various firing patterns of hypothalamic FRNs, which, after the initiation of food intake, can be sorted into four types: sharp increase (type I), slow increase (type II), sharp decrease (type III), and sustained decrease (type IV) of firing rates. The feeding-related firing response of FRNs was rigidly related to the duration of food intake and, to a less extent, associated with the type of food. The majority of these FRNs responded to glucose and leptin and exhibited electrophysiological characteristics of putative GABAergic neurons. In conclusion, our study demonstrated the diversity of neurons in the complex hypothalamic network coordinating food intake.

## Introduction

Neural coding of feeding behavior remained unknown for more than half a century due to technical difficulties in accessing deep hypothalamic structures for precise *in vivo* recording. Another obstacle is the heterogeneity and cross-modal signals of hypothalamic cells, which make it difficult to link cellular activity to specific behavior stimuli. Early studies revealed the lateral hypothalamus (LH) as the hunger center, because its lesion caused hypophagia (Anand and Brobeck, 1951; Grossman et al., 1978). In contrast, the electrical stimulation of LH induced hyperphagia (Hoebel and Teitelbaum, 1962; Margules and Olds, 1962). Later, it was shown that LH neurons respond to food intake (Hoebel and Teitelbaum, 1962; Schwartzbaum, 1988). Early studies employing *in vivo* recording showed that LH neurons encode sensory stimuli (Norgren, 1970; Yamamoto et al., 1989), including reward-associated cues (Nakamura et al., 1987). LH neurons also fire during both feeding (Burton et al., 1976; Schwartzbaum, 1988) and drinking (Tabuchi et al., 2002), as well as encode circadian rhythm (Ono et al., 1981), respond to odors (Shiraishi, 1988) and taste of glucose (Nakamura et al., 1989; de Araujo et al., 2006). However, due to limitations of the extracellular recording technique, none of these studies identified the chemical nature of feeding related neurons in the LH. Because many neuronal subpopulations co-exist in the LH, it is necessary to combine pharmacological and electrophysiological characteristics to link types of neural response with cell type in the LH.

The majority of LH neurons affecting feeding behavior are peptidergic in nature. There are three main neuropeptides in the LH, orexin/hypocretin (ORX/hcrt), melanin-concentrating hormone (MCH), and neurotensin (NT), which co-expressed with classical neurotransmitters in individual neurons to specifically modulate feeding behavior. ORX/hcrt neurons contain glutamate (Rosin et al., 2003; Torrealba et al., 2003), central administration of ORX/hcrt acutely promotes feeding (Sakurai et al., 1998) and their activity strongly correlates with finding novel food and its consumption (Mileykovskiy et al., 2005). ORX/hcrt neurons are activated in anticipation of feeding, couple arousal and feeding behaviors (Akiyama et al., 2004; Mieda et al., 2004). Interestingly, ORX promotes feeding during the day but not during dark phases (Haynes et al., 1999; Yamanaka et al., 1999; McGregor et al., 2011), indicating that ORX promotes feeding via an increase of arousal. In contrast to ORX/hcrt neurons, MCH neurons are GABAergic and MCH only amplifies consumption (e.g., size and amount; Baird et al., 2006, 2008), suggesting that MCH regulates general consumption behavior, but not necessarily hedonic aspects of feeding (Clegg et al., 2002; Sakamaki et al., 2005). Furthermore, MCH neurons also coordinate energy expenditure and metabolism (Rossi et al., 1997; Shimada et al., 1998; Marsh et al., 2002) and are able to sense nutrient status, such as glucose levels, and thus enhance the motivation to feed (Kong et al., 2010). The third neuropeptide, NT, is less studied. Several reports demonstrate that NT can co-express GABA or glutamate (Leinninger et al., 2011; Kempadoo et al., 2013). Its central infusion modestly decreases food intake in satiated and food-deprived rodents (Stanley et al., 1983; Vaughn et al., 1990; Boules et al., 2000) and promotes reward responding (Kempadoo et al., 2013) possible through inhibition of ORX/hcrt neurons (Goforth et al., 2014).

Activity of LH peptidergic neurons is largely dependent on nutritional and hormonal signals: glucose inhibits ORX/hcrt neurons (Muroya et al., 2001; Yamanaka et al., 2003), but excites MCH neurons (Burdakov et al., 2005), while leptin inhibits both types of cells (Goforth et al., 2014; Sheng et al., 2014). As for NT neurons, leptin activates them (Leinninger et al., 2011), although the effect of glucose was not studied in this neuronal type.

Despite accumulated knowledge of the LH neuronal types and circuits involved in food intake, the correlation of the *in vivo* activity of LH cells with actual feeding behavior is poorly studied. Importantly, the precise structure of firing patterns and neural dynamics of such feeding related neurons (FRNs) in behaving animals remains largely elusive. Using tetrode-based recording techniques, we report here the existence of four distinct types of hypothalamic neurons, which exhibit specific firing patterns directly related to the feeding behavior of freely moving mice.

## Materials and methods

### Behavioral protocols

#### Experimental animals

Twenty C57BL/6J male mice (3–8 months old) were used in the experiment. The animals were adapted in the animal facility after delivery from (shanghai SLAC laboratory animal co. ltd) for 4 weeks in a standard day/night cycle (12:12 h) room. Then the animals were relocated in separated plastic barrel homecages (55 cm in diameter, 42 cm in height; with nest/food/water), and were handled 5 min per day for a week. The animals were then implanted with chronic electrodes for *in vivo* recording. After recovery from the surgery, the animals were put into a dim recording chamber for 30 min per day, allowing them to get used to the recording environment and to find the clear signal of neurons via propelling electrodes gradually into the target area (1–4 weeks). After recording (usually over the course of several months), animals were sacrificed by overdose of isoflurane and transcardially perfused with 4% paraformaldehyde (pH 7.4). Brains were extracted and sectioned (thickness is 40 μm) followed by Nissl staining for verification of electrode placement in the hypothalamus. Miss-targeted animals were excluded from the analysis. All mouse work described in this study have been conducted according to Animals Act, 2006 (China) and approved by the Institutional Animal Care and Use Committee (IACUC approval ID #AR201404006) of the East China Normal University.

#### Recording sections include pre-test, test, and post-test

In all of the feeding related tests, the implanted mice were fasted for 24 h in homecages before feeding tests started and then they were placed in the recording chamber to get ready to record. All of the recordings were split into three sessions: (1) 30 min recording as pre-test baseline control, mouse can freely explore in the recording chamber; (2) 30 min recording during the feeding tests, mouse can approach the food and eat freely; during feeding tests the food was delivered automatically at a rate of 3–9 min per pellet, depending on the food amount (25, 50, and 100 mg). In each session food was delivered three to seven times; (3) 30 min recording after the feeding tests, the mouse can drink, explore and sleep in the recording chamber. Each feeding test was separated by a 1-day break to keep the mouse motivated for the feeding tests.

#### Recording of regular chow induced activity of FRNs

Chow pellet (weight of each pellet was 25 ± 5 mg) was used for the detection of FRNs. The food was delivered at a rate of 3 min per pellet, and each session contained seven times food delivery. The fasted mouse usually picked up the food within 15 s after delivery and continuously ate until it finished the whole pellet. Recordings of mice which didn't pick up food within 15 s or were disrupted during food intake were excluded from the data analyses.

#### Recording of activity of FRNs induced by intake of different amounts of food

In the food amount test, each chow pellet was cut and weighed. The food was delivered seven times with 3 min intervals (mean weight of each food pellet was 25 ± 5 mg in session 1), or five times with 5 min intervals (mean weight of each food pellet was 50 ± 10 mg in session 2), or three times with 9 min intervals (mean weight of each food pellet was 100 ± 10 mg in session 3). Each animal was sequentially processed through three sessions with the interval of 24 h to keep them motivated for food intake in each session.

#### Recording of activity of FRNs induced by intake of distinct food

The mice were subjected to two sessions: regular chow and cheese (mean weight of each pellet was 25 ± 5 mg), starting with regular chow, and each session was separated by 1 day.

### Electrophysiology

#### Implantation of chronic electrodes

Thirty-two channel chronic electrodes were hand-made, consisting of eight tetrodes and one specially designed microdrive. The microdrives were manually assembled as described previously (Lin et al., 2006). The tetrodes were made with Φ0.0005 inch nichrome wires (Stablohm 675, California Fine Wire Company, CA, USA). Eight tetrodes were loaded into the micro-drive via a guiding tube and were arranged in parallel order. Assembled electrodes were gold plated and impedance of each channel was measured between 250 and 350 kOhm. During implantation, surgery animals were anesthetized with 1.0% pentobarbital and then placed into a stereotaxic frame. Bregma position and horizontal level were aligned during implantation. Tetrodes tips were implanted above 0.5 mm of the hypothalamus and the microdrive was fixed on the skull by six screws and dental cement. Three to five days after the mice recovered from the surgery, tetrodes were propelled 100–200 μm per day into the target area (AP: −2.0 mm; ML: 2.0 mm, DV: 5.0 mm) followed by the *in vivo* recording.

#### *In vivo* recording and spike sorting

Video signal captured by CinePlex Behavioral Research System (Plexon, Inc., TX, USA) and electrophysiological signal acquired by Multichannel Acquisition Processor (MAP) Data Acquisition System (Plexon, Inc., TX, USA) were synchronized. NI USB-6501 Digital input/output board (National Instrument) served as feedback control of behavioral tests. The local field potentials were sampled at 1000 Hz with low pass filter while spike waveforms of AP were sampled at 40 kHz. Threshold of spikes was manually set in each channel to get the best noise-signal ratio. One thousand four hundred microseconds of whole spike duration with 200 μs pre-spike waveforms were sampled to capture the full AP waveform of hypothalamus unit. Offline Sorter (Plexon, Inc., TX, USA) was used for spike sorting manually after recording. Each waveform was aligned; principal component analyses (PCA) features were used to sort spike waveforms into separated clusters: units with refractory period larger than 1500 μs (<0.1% error) were accepted as one single neuron. Maximum 7 units were well-sorted on single tetrode in hypothalamus (see Supplementary Figure 2).

#### Data analysis

Neuroexplorer 3.2 was used to draw the rate histogram and perievent time histogram (PETH). MATLAB 2014b, Origin 9.0 and Sigmaplot 12.0 were used in statistical analyses. PETH was the main method to detect feeding response and analyze features of all FRNs. The y axis represents the average frequency of FRNs' response during five times of repetitive food intake, while the x axis represents the time scale ranging from −30 to 90 s of each feeding trial. The zero point of x axis was aligned with feeding start time by analysis of video recording. The spike time of AP is plotted in lines above each PETH, five rows indicate five repetitive trials. The feeding duration was defined by “feeding end time minus feeding start time.” The feeding start time was verified in videos of mice grasping food and starting to bite; the feeding end time was also verified in videos of mice finishing eating and starting to move (mice usually stand still during feeding). The response duration of FRNs was defined by response offset (sec) minus response onset (sec), where the response onset and offset were the time point when AP frequency was over three times the standard deviation (99% confidence) of baseline firing rate.

#### Response separation of feeding related neurons

We characterized each type of FRNs based on a combination of the three parameters in PETH (peak response, peak latency, and peak decay). The peak response was calculated as the maximum or the minimum frequency after feeding start minus baseline firing rate, this may vary in different types of FRNs, e.g., Type I and II FRNs' maximum frequency was higher than the baseline firing rate, thus the peak response was positive; Type III and IV FRNs' peak response was negative because the baseline firing rate was usually higher than the frequency during food intake, thus the minimum frequency was used in calculation; the latency was defined as the time point of maximum frequency of response plus the response onset, then divided by the firing duration. The latency differentiated the fast response from the slow response in initiation of food intake. The decay was calculated as the half decay response divided by the peak response, and the half decay response was taken the AP frequency at middle of response peak time and offset time, then minus baseline firing rate; the decay separated the sustainable discharge property of FRNs during food intake.

Two parameters were used to distinguish GABAergic neurons from principal cells in FRNs: Trough to peak duration of the extracellular spike waveforms was calculated by the time of peak after trough minus the trough time (**Figure 6B**). Baseline firing rate was defined by the number of spikes divided by the time (seconds) in pre-feeding control section. Both trough to peak duration and baseline firing rate were widely used in several *in vivo* recording studies. (Barthó et al., 2004; Vigneswaran et al., 2011; Tsunada et al., 2012) We employed two thresholds in baseline firing rate (5 Hz) and trough-to-peak duration (0.42 ms) to divide FRNs into putative glutamatergic neurons, putative GABAergic neurons and undetermined neurons (Barthó et al., 2004; Ison et al., 2011).

### Pharmacology

Mice subjected to regular diet were placed in the empty recording chamber and recorded for 1 h at basal state. After they were i.p. injected with vehicle (0.9% saline; 10 ml/kg), 2-D-Glucose (Sigma; 2 g/kg b.w.), and leptin (ProSpec Inc.; 1 mg/kg b.w.) and recorded for 2 h without any interruptions. Each animal underwent all sessions with injection of saline, glucose and leptin, separated by 1 day. In the pharmacology test, the response was defined as two times standard deviation of baseline response, average AP frequency of 1 h pre-injection is the baseline response, and the average AP frequency of 1 h post-injection was used to calculate the response of glutamate or leptin.

## Results

### Four types of feeding related neurons in the hypothalamus

In twenty mice implanted with electrodes, the spiking activities of 509 well-sorted hypothalamic units were recorded (Supplementary Figures 1, 2). After detect feeding related neural response in perievent time histogram (PETH, 99% confidence), the cells with an absolute value of peak response (see Methods) higher than 6 Hz were characterized as feeding related neurons (FRNs). In total, 50 cells were selected as FRNs from all recorded units recorded (9.8%, 50/509; Supplementary Table 1). These FRNs were further subdivided into four groups based on their peak response, peak latency and peak decay (Methods and Table 1).

**Table 1:** **Statistical features of all types of FRNs**.

	**Type I**	**Type II**	**Type III**	**Type IV**	**Other cells**
Cell number	15	14	9	12	459
DG+	1	4	4	2	28
DG−	4	2	2	4	24
DG N/A	1	1	2	2	88
Leptin+	0	1	1	0	20
Leptin−	3	4	3	4	13
Leptin N/A	1	1	1	2	149

**Figure 1:**
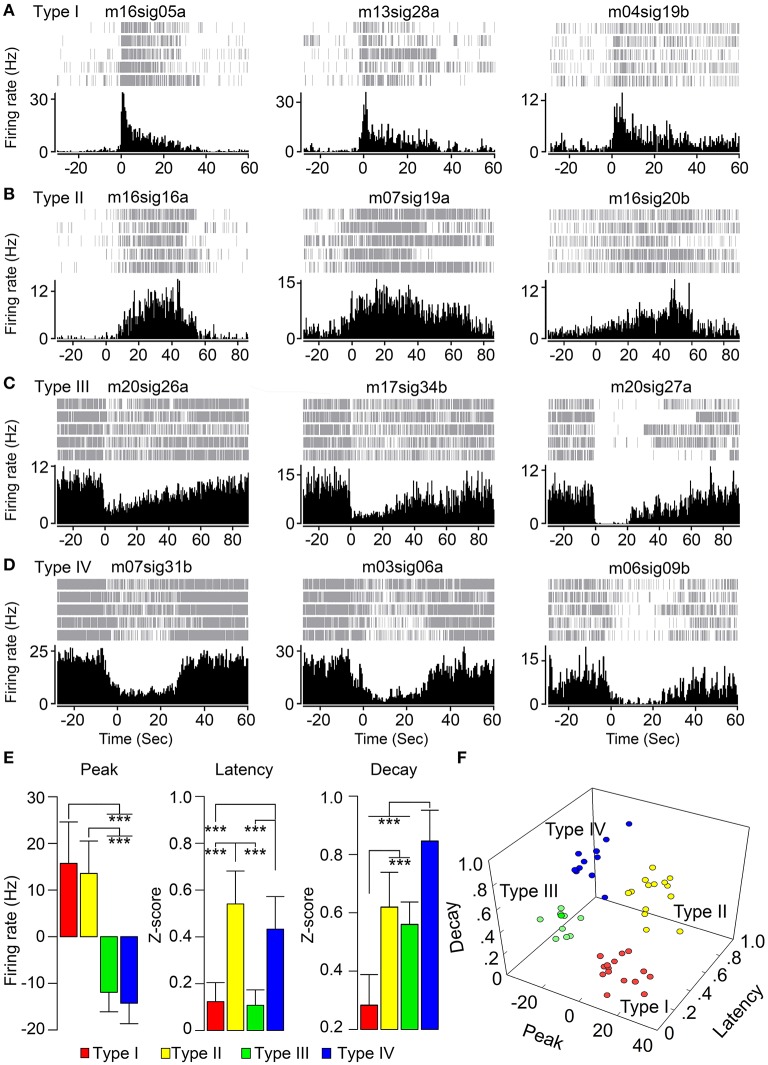
**Distinct feeding related firing patterns of hypothalamic neurons. (A)** Type I of FRNs with the sharp increase of firing rate at beginning of food intake. **(B)** Type II of FRNs with an increase of firing rate. **(C)** Type III of FRNs with the sharp decrease of firing rate. **(D)** Type IV of FRNs with sustain decrease of firing rate during feeding. **(E)** Feeding related firing patterns of FRNs were compared by three parameters of their perievent time histogram: peak response, peak latency and peak decay (^***^p < 0.001, multiple *t*-test with Bonferroni correction). **(F)** Four types of FRNs were separated in 3D scatters graph.

Type I of FRNs showed a sharp increase of firing rate at the beginning of feeding, then a decrease of firing despite continuous food intake (Peak Response = 15.7 ± 8.9 Hz, Peak Latency = 0.12 ± 0.08, Peak Decay = 0.28 ± 0.11, *n* = 15 neurons, Figure 1A). Type II of FRNs was characterized by a slow increase of firing during food intake and sustained firing until the end of food intake (Peak Response = 13.6 ± 6.9 Hz, Peak Latency = 0.54 ± 0.14, Peak Decay = 0.62 ± 0.12, *n* = 14 neurons, Figure 1B). Type III of FRNs demonstrated sudden inhibition at the beginning of feeding, then recovered quickly during feeding (Peak Response = −11.9 ± 4.2 Hz, Peak Latency = 0.11 ± 0.07, Peak Decay = 0.56 ± 0.08, *n* = 9 neurons, Figure 1C). Type IV of FRNs had gradual decrease of firing and remained silent during feeding (Peak Response = −14.3 ± 4.1 Hz, Peak Latency = 0.43 ± 0.15, Peak Decay = 0.85 ± 0.11, *n* = 12 neurons, Figure 1D). Based on the direction of responses, types I and II can be seen as “ON” response group, while types III and IV as “OFF” response group. The Figure 1E show the average values of the parameters described above for each population of FRNs, and there were significant differences among each group (see *P*-value of *t*-test in Table 2). After feature projection of those characters of all recorded FRNs into the 3D coordinate system, four unique clusters clearly emerged (Figure 1F).

**Table 2:** The ***p***-values of multiple ***t***-tests with Bonferroni correction for all possible combinations between FRNs types, food amount, and food type (^***^p < 0.001).

**Groups combination**	**Refer to**	**Peak**	**Latency**	**Decay**
Type I–II	Figure 1E	0.85	1.6 e-9(^***^)	7.8 e-8(^***^)
Type I–III	Figure 1E	2.0 e-9(^***^)	0.93	6.2 e-7(^***^)
Type I–IV	Figure 1E	1.9 e-8(^***^)	2.2 e-6(^***^)	2.2 e-12(^***^)
Type II–III	Figure 1E	9.0 e-9(^***^)	6.3 e-9(^***^)	0.39
Type II–IV	Figure 1E	2.1 e-9(^***^)	0.17	1.9 e-4(^***^)
Type III–IV	Figure 1E	0.79	3.5 e-6(^***^)	2.8 e-6(^***^)
ON: 25–50 mg	Figure 3A	0.14	0.60	0.99
ON: 50–100 mg	Figure 3A	0.50	0.62	0.94
ON: 25–100 mg	Figure 3A	0.72	0.99	0.96
OFF: 25–50 mg	Figure 3C	0.21	0.45	0.42
OFF: 50–100 mg	Figure 3C	0.02	0.99	0.10
OFF: 25–100 mg	Figure 3C	0.03	0.88	0.58
ON: cheese-control	Figure 4C	0.31	0.94	0.14
OFF: cheese-control	Figure 4C	0.41	0.83	0.22

**Figure 2:**
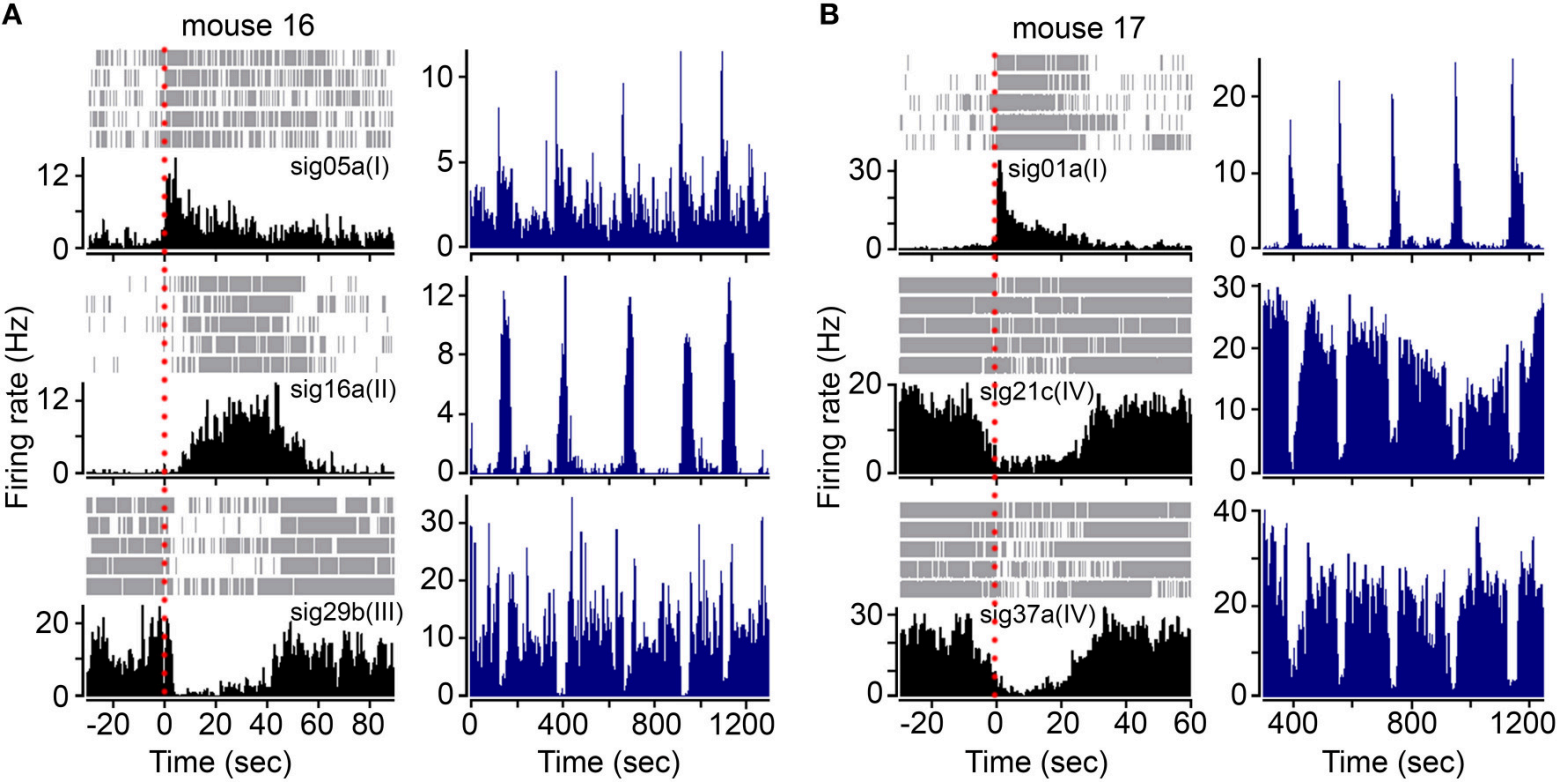
**FRNs orchestrate during food intake in type specific pattern. (A)** Three simultaneously recorded different types of FRNs (type I, II, and III) during feeding behavior in one mouse. **(B)** Three simultaneously recorded different types of FRNs (type I, IV, and IV) during feeding behavior in another mouse. Onset of feeding was aligned by red dashes in PETH (left panel). Type I and III FRNs peaked exactly at the beginning of food intake, while type II and IV respond slowly and peaked in the middle of food intake.

The FRNs of different types (I, II, III, and IV) simultaneously responded to the beginning of food intake, although the latency of response onset may vary between different types (Figure 2). Here, we listed three different types of FRNs simultaneously recorded in two animals: type I, II, and III in mouse 16 (Figure 2A), type I, IV, and IV in mouse 17 (Figure 2B). Both PETHs (Figures 2A,B; left panel) and rate histograms (Figures 2A,B; right panel) exhibit synchronized firing patterns among different types. When we aligned the timing of food intake in PETHs, we found that type IV FRNs often respond when the animal aware the food was delivered (before the animal starts to eat), type I and III FRNs respond exactly at the time the animal takes the first bite, and finally a late response can be found in type II FRNs within few seconds after feeding started. Importantly, all these type of FRNs were firing robustly during feeding, and their basal firing rate remained relatively stable in non-feeding periods, including exploratory behavior and rest.

### Response of feeding related neurons correlates with food amount

**Figure 3:**
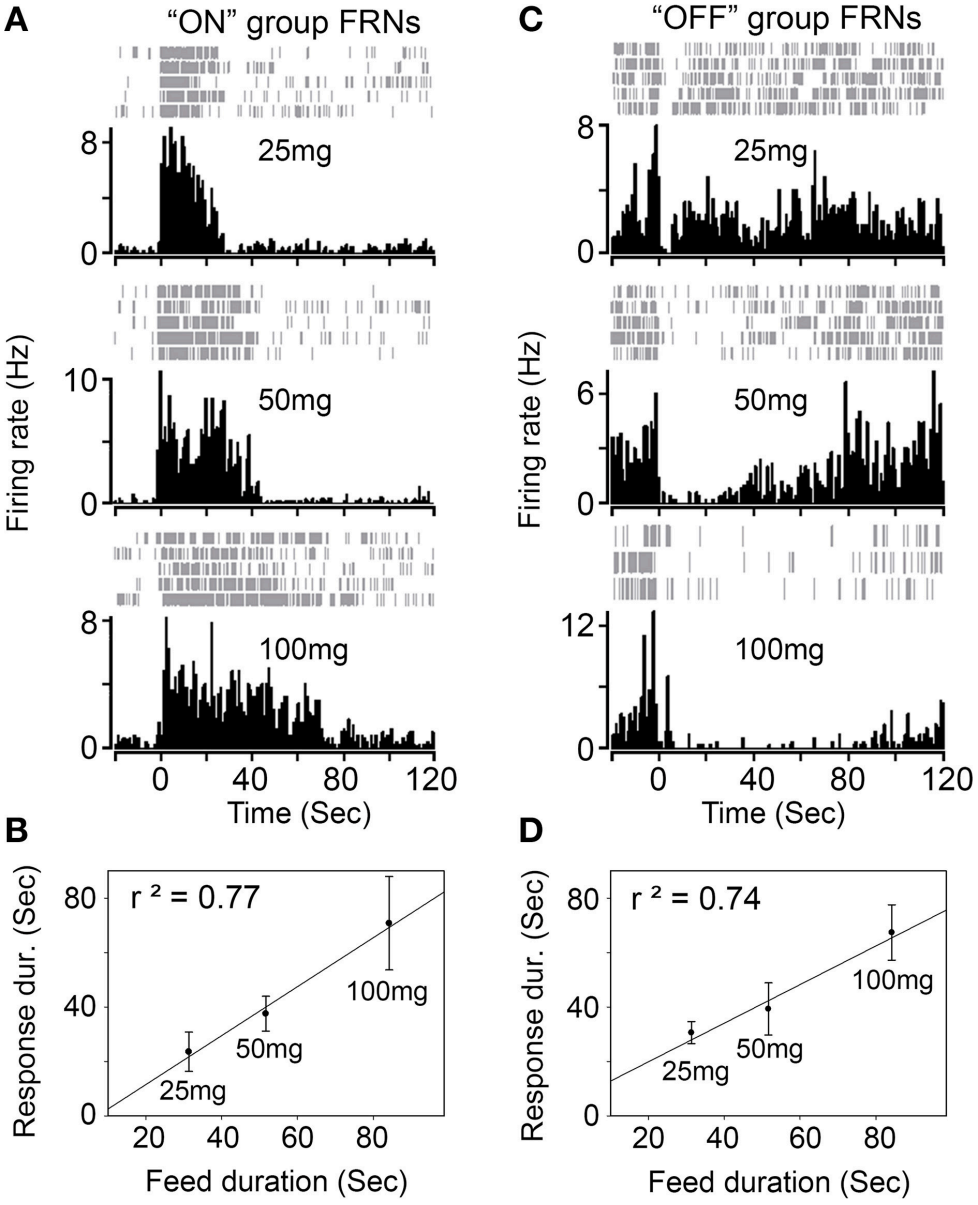
**Response of FRNs to the amount of food. (A)** One example of “ON” group FRN was tested with three different amount of chew food (25 mg, 50 mg and 100 mg). **(B)** Firing response of “ON” group FRNs correlated with food amount and feed duration (*n* = 21 neurons, *N* = 8 mice). **(C)** One example of “OFF” group FRN was tested with three different amount of chew food (25, 50, and 100 mg). **(D)** Firing response of “OFF” group FRNs correlated with food amount and feed duration (*n* = 17 neurons, *N* = 8 mice).

Next, we tested whether different amounts of food (25, 50, and 100 mg) affect the firing properties of FRNs. We recorded the typical response of “ON” (types I and II) and “OFF” group of FRNs among the three food amount trials (Figure 3). On average, the feeding duration increased with food amount: 31 ± 6 s after intake of 25 mg, 52 ± 4 s after intake of 50 mg and 84 ± 15 s after intake of 100 mg (*N* = 8 mice). In parallel, the firing duration of FRNs also increased with food amount: 24 ± 7 s (25 mg), 38 ± 6 s (50 mg), 70 ± 17 s (100 mg) in the “ON” group of FRNs (Figure 3B, *n* = 21 neurons), and 31 ± 4 s (25 mg), 39 ± 10 s (50 mg), 67 ± 10 s (100 mg) in the “OFF” group of FRNs (Figure 3D, *n* = 17 neurons). Linear regression showed that excitation or inhibition response of FRNs were closely related to food amount as well as feeding duration, both in the “ON” (*r*^2^ = 0.77) and “OFF” group (*r*^2^ = 0.74). In contrast, peak response, peak latency and peak decay of both “ON” and “OFF” groups remain unchanged in the three food amount tests. (see *p*-value of *t*-test in Table 2).

### Response of feeding related neurons is not affected by food types

**Figure 4:**
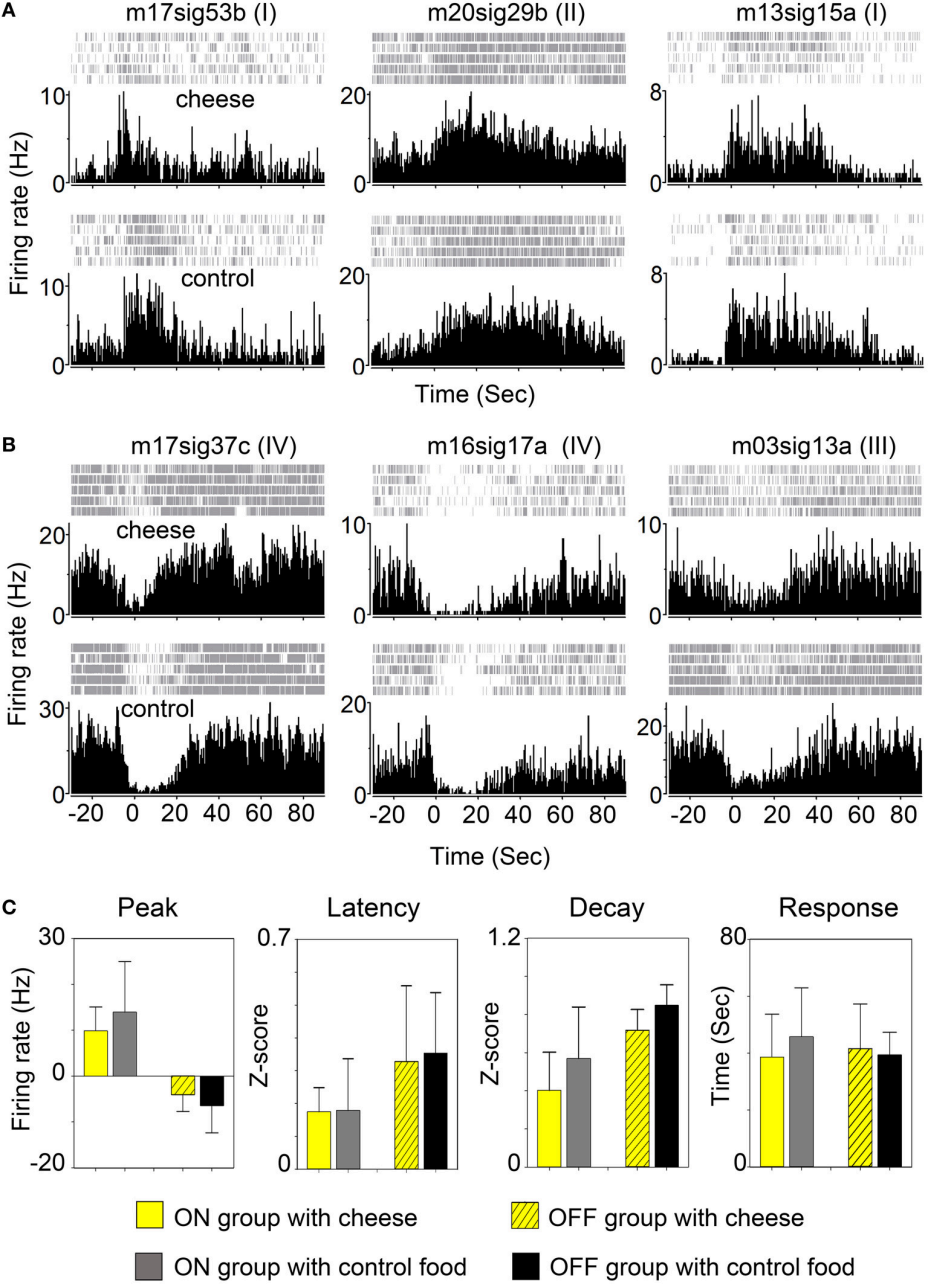
**Response of FRNs to distinct food type. (A)** Three “ON” group FRNs in cheese test (top panels) and control chow food test (bottom panels). **(B)** Three “OFF” group FRNs in cheese test (top panels) and control chow food test (bottom panels). **(C)** Statistical data showed the variations of peak response, peak latency, peak decay and firing duration between cheese and chow food in both ON group (*n* = 9 neurons, *N* = 6 mice) and OFF group (*n* = 7 neurons, *N* = 6 mice). No significant difference among any parameter in each group was found.

We then asked whether activity of FRNs depends on specific food types. We recorded FRNs neurons exposed sequentially to regular chow and cheese. As can be seen in the comparison of either three “ON” group FRNs (Figure 4A) and three “OFF” group FRNs (Figure 4B), firing patterns between regular chow and cheese revealed that neither peak response, peak latency, peak decay nor response duration had any difference either in the “ON” (Figure 4C, *n* = 9 neurons, *N* = 6 mice) or “OFF” (Figure 4C, *n* = 7 neurons, *N* = 6 mice) group of FRNs (see *p*-value of *t*-test in Table 2).

### Effects of glucose and leptin on feeding related neurons

Next, we asked whether recorded hypothalamic cells are sensitive to nutritional and hormonal signals—glucose and leptin. In 8 mice, 140 units were tested for glucose and 182 units were tested for leptin. Among them 37% (52/140) units responded to glucose: 20% (28/140) were activated and 17% (24/140) were inhibited, while only 18% (33/182) units responded to leptin: 7% (20/182) were activated and 11% (13/182) were inhibited (Table 1).

**Figure 5:**
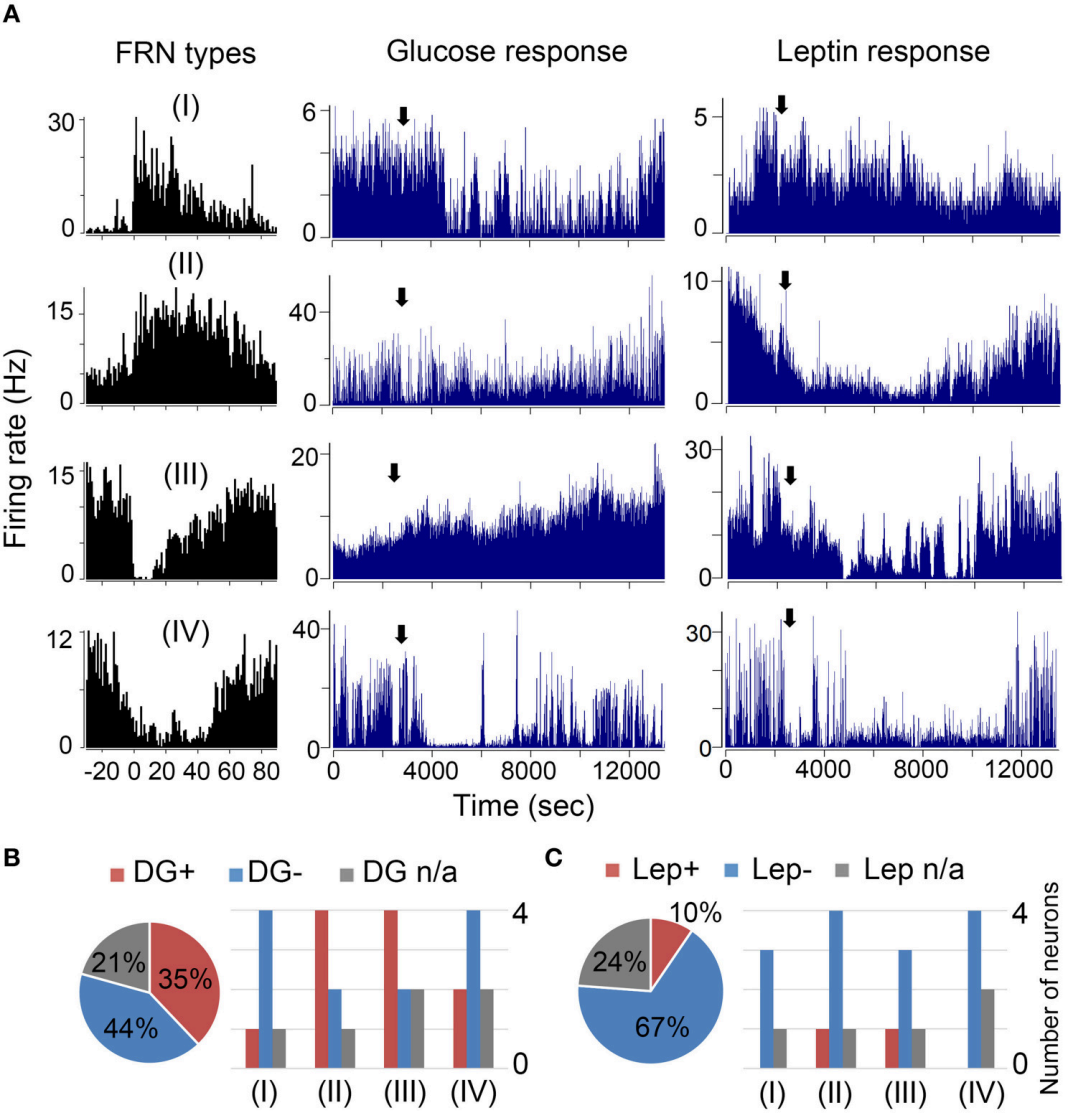
**Effects of glucose and leptin on FRNs. (A)** Four examples of each type FRNs respond to glucose and leptin. In first line type I FRN's spike rate decreased after glucose and leptin i.p. delivery. In second line type II FRN's spike rate increased after glucose delivery but decreased after leptin i.p. delivery. In third line type III FRN's firing rate decreased after glucose i.p. delivery but increased after leptin i.p. delivery. In fourth line type IV FRN's firing rate decreased both after glucose and leptin i.p. delivery. **(B)** Left panel was the ratio of all FRNs' response to glucose (*n* = 29 neurons, *N* = 8 mice), right panel is statistical results of each type FRNs' response to glucose. Similarly, the statistics of FRNs' response to leptin were showed in left panel **(C)** was the ratio of all FRNs' response to leptin (*n* = 21 neurons, *N* = 8 mice) while the right panel was statistical results of each type FRNs respond to leptin.

Further, we asked whether the recorded FRNs are sensitive to glucose and leptin. Due to the instability of extracellular signals and cell death in chronic recording condition, only parts of the FRNs were tested: 29 FRNs were tested for glucose and 21 FRNs were tested for leptin (*N* = 8 mice). We found that 79% (23/29) of FRNs responded to glucose, either by an increase (11/29) or decrease (12/29) of firing rates (Figure 5A middle panel), while 21% (6/29) of FRNs did not respond to injection of glucose. Similar results were obtained after the injection of leptin: 76% (16/21) of FRNs responded to leptin, among which 67% (14/21) of FRNs decreased firing rate, 24% (5/21) of FRNs did not change their firing rate and only 10% (2/21) of FRNs increased firing rate (Figure 5A; right panel).

Ruled out the none response cells, majority Type I were inhibited by both glucose (4/5) and leptin (3/3); and Type II were activated by glucose (4/6) but inhibited by leptin (4/5); most of Type III were also activated by glucose (4/6) and inhibited by leptin (3/4); majority Type IV were inhibited by both glucose (4/6) and leptin (4/4) (Figures 5B,C). We did not find any difference in FRNs' firing rate after giving saline.

### Electrophysiology character shows FRNs are mostly putative GABAergic neurons

**Figure 6:**
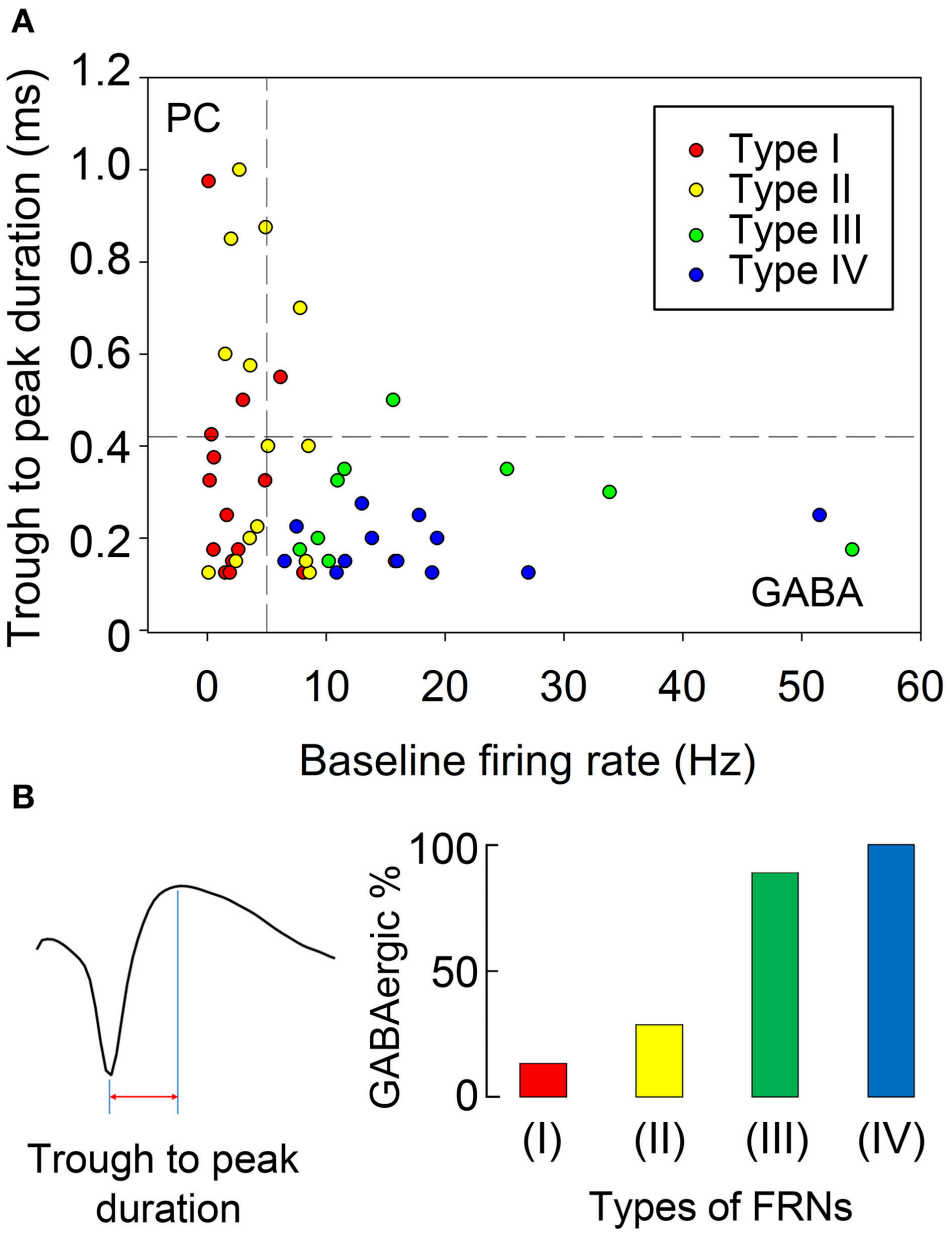
**Discrimination of putative GABAergic and glutamatergic FRNs. (A)** We set the trough to peak threshold of 0.42 ms and baseline firing rate threshold 5 Hz to separate scatters into four quadrants. Putative glutamatergic neurons were in quadrant II (TPD > 0.42 ms, BFR < 5 Hz), putative GABAergic neurons were in quadrant IV (TPD < 0.42 ms, BF R> 5 Hz) and undetermined cells are in quadrant I and III. 26 FRN were set as putative GABAergic neurons and 8 FRNs were set as putative glutamatergic neurons, remaining 16 FRNs were set as undetermined cells. **(B)** Trough to peak duration of FRNs' extracellular spike waveform was showed as example in the left. Composite of each type of FRNs were showed in the right, each color represented GABAnergic neuron in each type FRNs. Type I & II FRNs had a mixed composition of putative glutamatergic and GABAergic neurons, although 66% type I and 36% type II remain undetermined. 89% type III and 100% type IV FRNs belonged to putative GABAergic neuron.

The baseline firing frequency and the spike duration were two well-established criteria to separate GABAergic neurons from glutamatergic neurons (Buzsáki and Vanderwolf, 1983; Singer and Gray, 1995; Skaggs and McNaughton, 1996). We used trough to peak duration (TPD, criteria 1, Figure 6B) and baseline firing rate (BFR, criteria 2) to sort all recorded FRNs plotted in 2D scatters. Then we set the trough to peak threshold to 0.42 ms and baseline firing rate threshold to 5 Hz (Barthó et al., 2004; Ison et al., 2011; Peyrache et al., 2015), and separate scatters into four quadrants. The classifications based on both criteria were shown in Figure 6A. 2D plot of trough to peak duration and baseline firing rate exhibit at least two groups of FRNs: (1) putative GABAergic neurons with TPD < 0.42 ms and BFR > 5 Hz (Figure 6A, quadrant IV); (2) putative principal cells which were commonly considered as glutamatergic neuron with TPD > 0.42 ms and BFR < 5 Hz (Figure 6A, quadrant II). Comparing characteristics of four types of FRNs with their firing pattern, we found that 28 out of 50 FRNs have characteristics of putative GABAergic neurons and 8 of them have characteristics of putative principal cells, other 14 remain vague between GABAergic neurons and principal cells. As a conclusion of separation: In 15 type I FRNs (2 GABA, 3 PC, 10 undetermined); in 14 type II FRNs (4 GABA, 5 PC, 5 undetermined); in 9 type III FRNs (8 GABA, 1 undetermined); in 12 type IV FRNs (12 GABA) (Figure 6B).

## Discussion

### Four types of FRNs

The LH is composed of heterogeneous groups of cells, often expressing various neuropeptides concomitantly with GABA or glutamate. In our study, we identified hypothalamus FRNs, which were classified into four functional types based on peak direction, latency and duration of responses. Types I and II FRNs were activated during food intake (increased firing rate with fast or slow latency respectively), representing the “ON” group of FRNs. On the contrary, types III and IV were inhibited during feeding (decreased firing rate or total cessation of firing), therefore representing the “OFF” group of FRNs. Further, subtypes in the “ON” group were separated by onset of peak response, type I with immediate onset of discharge to the maximum frequency, while type II with delayed discharge and gradually reaching of maximum frequency. The “OFF” group of FRNs can be separated by decay value of response, type III with fast decay of discharge inhibition and type IV with long decay of discharge inhibition. These four types of FRNs seem to encode all of the basic properties of feeding behavior in the LH.

It is interesting that some types of FRNs respond “prior” to feeding behavior compared to other types: e.g., type IV FRNs decrease firing rate and some type II FRNs has an instant burst firing at the moment when the animal saw the food. We suspect that this pre-feeding neural activity may correlate with alert or arousal response. From the shape of the neural response, we hypothesize that type I and type III were paired in encoding the initiation of food intake (e.g., bite and taste), while type II and type IV may serve to sustain the feeding process (e.g., swallow and digest).

### FRNs activity depends on duration of food intake

We found that increasing the amount of the meal gradually increases the response duration of FRNs, but not the peak frequencies or the other features of responses. These results suggest that FRNs' response basically follows the duration of mice eating. The “ON” group of FRNs increases firing after feeding start and ceases firing before feeding ends. The “OFF” group of FRNs decreases firing during the feeding process and slowly recovers after feeding ends. The characters of response (onset, peak) remain unchanged as the meal increased, which indicates the specificity of response was robust in each type of FRNs.

We tested the FRNs' response to different food types, and found no significant difference between cheese and chow food groups in either the same neuron or in cell populations (Figure 4). In some trials, we used chocolate, but we still found no obvious difference compared with cheese or chow pellet. To our observation, few FRNs respond to the sound of food delivery, more like an alert or arousal response, which was similar to response of orexin neurons (Mileykovskiy et al., 2005). Indeed, there are other stimuli which may activate LH neurons, like the taste of food (Burton et al., 1976; Schwartzbaum, 1988). In our study, we excluded neurons that responded to sound or smell, but did not respond to food intake. We also tested false food as a control object to real food, but the mice refused to approach fake food after being cheated two or three times, thus no robust response of the cell is recorded, although in the first or second trial, the initial response of fake food is similar to real food. Until animals recognize the object as food, they will not start to bite, thus it is hard to detect FRNs respond to non-food object. The FRN response during sleep remains constant; the “ON” group of FRNs' baseline firing rate was very low across all situations, besides food intake, and the “OFF” group of FRNs' baseline firing rate was high when awake, but decreased firing rate or changed into phasic firing during sleep. Our results are in accordance with the studies done by other researchers (Hassani et al., 2009, 2010).

Regarding the circadian rhythm of FRN's activity, we continuously recorded FRNs for 72 h to demonstrate if there is a circadian clock in the firing rhythm. Indeed, we found that there is higher discharge in the evening when the animal usually gets up to eat; the discharge lasts for about 2 h and then goes back to baseline and stays constant during rest hours. Another important issue is that social isolation can alter the HPA axis as well as affect the neural activity (Serra et al., 2005; Agís-Balboa et al., 2007). Therefore, we cannot exclude the effects of stress on both food intake and excitability of recorded neurons. However, individual housing of mice was only possible in our experimental setup because microdrives with electrodes on the head of each mouse are extremely fragile and co-housed mice can easily damage them.

From observations described above, we concluded that the firing of FRNs is more dependent on food intake duration rather than on types of food. Although, the baseline firing of FRNs may change with circadian rhythm, the response of FRNs in food intake is much stronger.

### Majority recorded FRNs are glucose and leptin sensing neurons

Nutritional and hormonal signals, such as glucose or leptin, led to opposite responses of FRNs to food intake (Figure 5). More than half of the FRNs were activated by glucose, and over one-third of the FRNs were inhibited, while most FRNs were inhibited and only few were activated by leptin. However, if we ruled out the FRNs and see all the other recorded cells in the hypothalamus, this ratio dropped much lower. Only 20% were activated and 17% were inhibited by glucose. Comparing the ratio of glucose response recorded in all cells in the hypothalamus, we can infer that the majority of FRNs are glucose-sensing neurons (Oomura et al., 1974). The four types of FRNs' pharmacology response were not equally distributed (Figures 5B,C) which suggests that each type may have their unique chemical features and receptors. Interestingly, the “ON” group contains more FRNs activated by glucose, while the “OFF” group is composed of cells that were preferentially inhibited by glucose. This finding may indicate bidirectional regulation of energy balance by the “ON” and “OFF” groups of FRNs.

The high number of FRNs responding to leptin suggests that the majority of FRNs expresses the leptin receptor (Figure 5C). The different distribution among types also implies a bidirectional network of body weight regulation (e.g., more FRNs were inhibited rather than activated by leptin). The majority of FRNs responded to nutritional and hormonal signals, indicating that they play an important role in energy balance and food intake regulation; however, the “biased” distribution of inhibition and activation response among four types of FRNs suggests that there may be a dominant type of cells in each type of FRN. The “ON” group possibly consists of peptidergic cells, which are inhibited by glucose, and the “OFF” group of FRNs can be inhibitory neurons, expressing leptin receptors. The results fit our finding that 40% of the GABAergic neurons in the LH are inhibited by glucose (Marsh et al., 2002; Karnani et al., 2013).

### Are FRNs GABAergic or glutamatergic neurons?

According to the high mean firing rates and narrow duration of action potentials (APs), the majority of FRNs appear to be putative GABAergic neurons, which is supported by recent findings (Karnani et al., 2013; Jennings et al., 2015). Another small part of FRNs is putative glutamatergic neurons representing the main type of neuropeptidergic neurons, and their firing pattern resembles random firing of neurons in the ventromedial nucleus (Sabatier and Leng, 2008).

Baseline firing rate is a key parameter to define GABAergic neurons. Although, peak response can be the same during feeding, GABAergic neurons have a much higher basal firing rate than pyramidal cells (Ison et al., 2011). Since types III and IV of FRNs have a relatively high baseline firing rate and narrow spike durations, the most of these two types FRNs are likely to be GABAergic neurons. (Figure 6).

Type I of FRNs has an acute response on feeding, and most of them were inhibited by both glucose and leptin. Furthermore, at least half of FRNs exhibited the same AP waveform and baseline firing similar to glutamate neurons. Based on these two features, we speculate that Type I FRNs are orexinergic neurons (Mileykovskiy et al., 2005) activated during feeding behavior (Akiyama et al., 2004; Mieda et al., 2004).

Type II of FRNs were activated by glucose and inhibited by leptin, and their electrophysiology parameters represented both GABA and glutamatergic neurons. This mixed composition is likely reflects the nature of neuropeptide neurons as they can simultaneously express GABA (van den Pol et al., 2004) and glutamate (Chee et al., 2015).

The “OFF” group of FRNs is characterized by narrow waveform, high basal firing rate, and inhibition by glucose and activation by leptin. Based on inhibitory responses during feeding, we assume that at least a part of the “OFF” FRNs were LepR expressing GABAergic neurons, as was reported previously (Karnani et al., 2013).

### Incorporation of FRNs in LH network controlling food intake

Our study revealed the presence in the LH of “ON” and “OFF” FRNs, which can be bidirectional modulated during food intake. As a possibility, the initiation of feeding behavior can be induced by “ON” cells, which can be reciprocally inhibited by “OFF” cells, receiving extra LH inputs (Elias et al., 1999; Goforth et al., 2014). However, precise interactions of FRNs within the local LH network to control feeding behavior, metabolism, and energy consumption remains further research.

## Conclusion

This study found “ON” and “OFF” feeding related neurons in mouse hypothalamus, further divided into four types, which fired synchronously to presumably orchestrate feeding behavior. The activity of FRNs was correlated with food amount, but not food type, and most of them were putative GABAergic neurons. Based on electrophysiological characteristics, electrodes location and sensitivity to glucose and leptin, we propose that FRNs are glucose sensitive neurons, expressing LepR. Incorporation of these groups in the network and their contribution to distinct forms of feeding behavior remains a subject of further research.

## Author contributions

YT, LL designed the experiments. YT performed surgery and experiments. YT, DB, and LL performed analysis. YT, VG, and LL wrote the manuscript. All of the authors discussed the results at all stages of the project.

### Conflict of interest statement

The authors declare that the research was conducted in the absence of any commercial or financial relationships that could be construed as a potential conflict of interest.
