# Supplementary material for: Distinct Types of Feeding Related Neurons in Mouse Hypothalamus
Source: Front Behav Neurosci. 2016 May 18;10:91. doi: 10.3389/fnbeh.2016.00091 (PMC4870269; doi:10.3389/fnbeh.2016.00091)
Supplement: Supplementary file 1 [file DataSheet1.docx]

Supplementary Material

Distinct types of feeding related neurons in mouse hypothalamus

Yan Tang^1, 2^, Diego Benusiglio^2^, Valery Grinevich^2^, Longnian Lin^1,*^

^1^Key Laboratory of Brain Functional Genomics (Ministry of Education and Shanghai), Institute of Brain Functional Genomics, School of Life Science and the Collaborative Innovation Center for Brain Science, East China Normal University, Shanghai 200062, China

^2^Schaller Research Group on Neuropeptides at German Cancer Research Center (DKFZ), Central Institute of Mental Health, and Cell Networks Cluster of Excellence at the University of Heidelberg, Heidelberg, Mannheim, Germany

***Correspondence:**

Longnian Lin, PhD

Institute of Brain Functional Genomics

East China Normal University,

3663 Zhongshan Road N.,

Shanghai, China 200062

Phone: [+86-21-62233533](tel:%2B86-21-62233533)

Fax: [+86-21-62601953](tel:%2B86-21-62601953)

E-mail: [lnlin@brain.ecnu.edu.cn](mailto:lnlin@brain.ecnu.edu.cn)

# Supplementary Figure Legends:

**Supplementary Figure 1.** Nissl-stained coronal sections showing tetrode positions in four animals. Positions of tetrode tracks are indicated by red dot. Analysis revealed that 85% of cases (17/20) tetrode positions were placed in the lateral hypothalamus as can be seen by implantation track.

**Supplementary Figure 2.** Spike sorting of recorded hypothalamic neurons

Left panel represents cells clusters recorded in the hypothalamus using offline sorting software, maximum 7 units can be sorted in one tetrode). X axis: PC1 EL1, Y axis: PC1 EL2, Z axis: PC1 EL4. Different colors clusters represent every single unit after sorting. Right panel is the waveforms of each cluster after sorting.

**Supplementary Table 1.** FRNs recorded in the hypothalamus

| Animals | neurons recorded | FRNs detected |
| --- | --- | --- |
| mouse 1 | 8 | 1 |
| mouse 2 | 12 | 1 |
| mouse 3 | 17 | 2 |
| mouse 4 | 24 | 3 |
| mouse 5 | 22 | 2 |
| mouse 6 | 26 | 3 |
| mouse 7 | 34 | 5 |
| mouse 8 | 20 | 3 |
| mouse 9 | 15 | 2 |
| mouse 10 | 20 | 2 |
| mouse 11 | 24 | 2 |
| mouse 12 | 15 | 0 |
| mouse 13 | 31 | 2 |
| mouse 14 | 12 | 0 |
| mouse 15 | 33 | 3 |
| mouse 16 | 56 | 5 |
| mouse 17 | 65 | 6 |
| mouse 18 | 22 | 2 |
| mouse 19 | 30 | 3 |
| mouse 20 | 23 | 3 |
| SUM | 509 | 50 |
