# Supplementary figures and images for: Distinct Types of Feeding Related Neurons in Mouse Hypothalamus
Source: Front Behav Neurosci. 2016 May 18;10:91. doi: 10.3389/fnbeh.2016.00091 (PMC4870269; doi:10.3389/fnbeh.2016.00091)

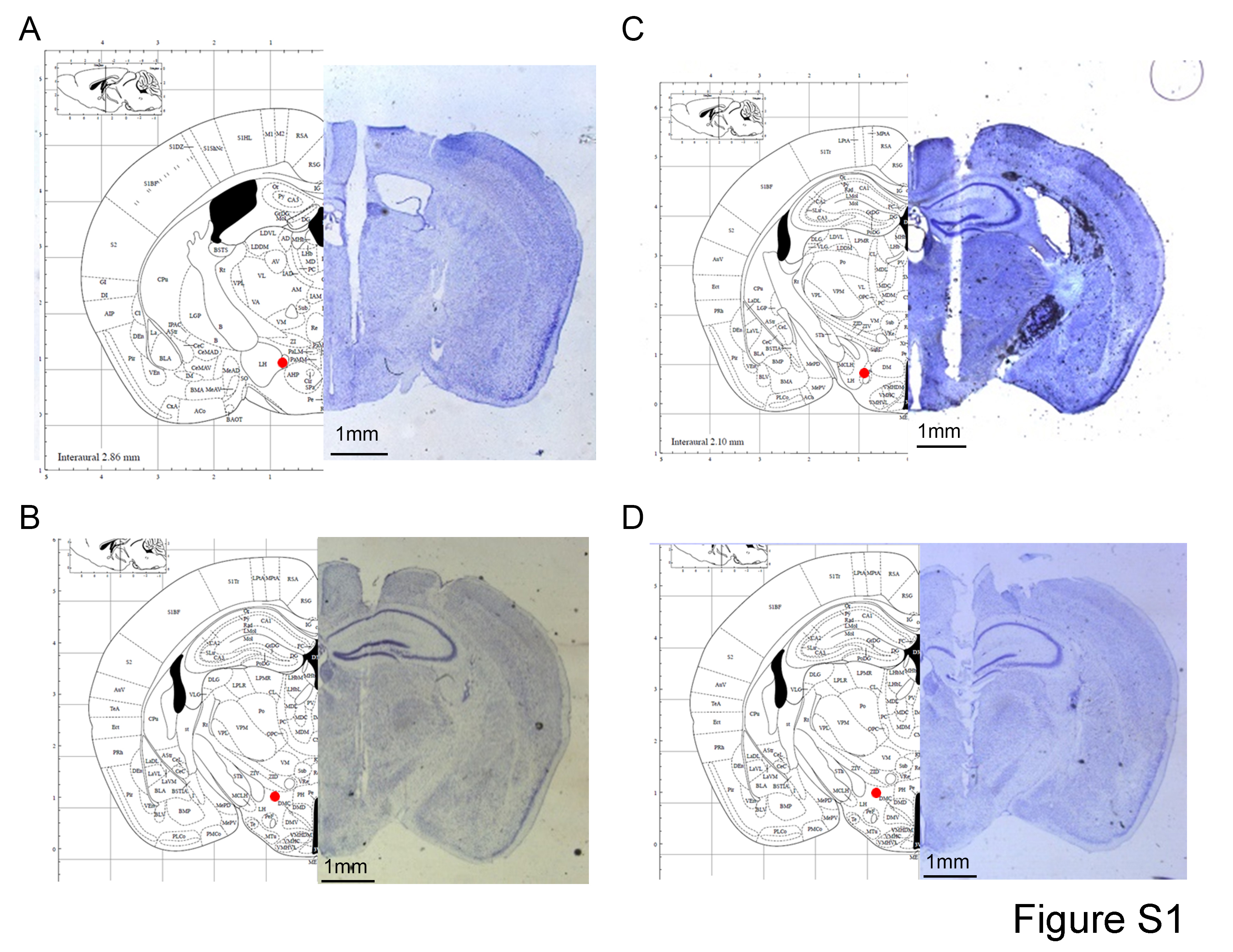

Supplement: Supplementary file 2 [file Image1.JPEG]

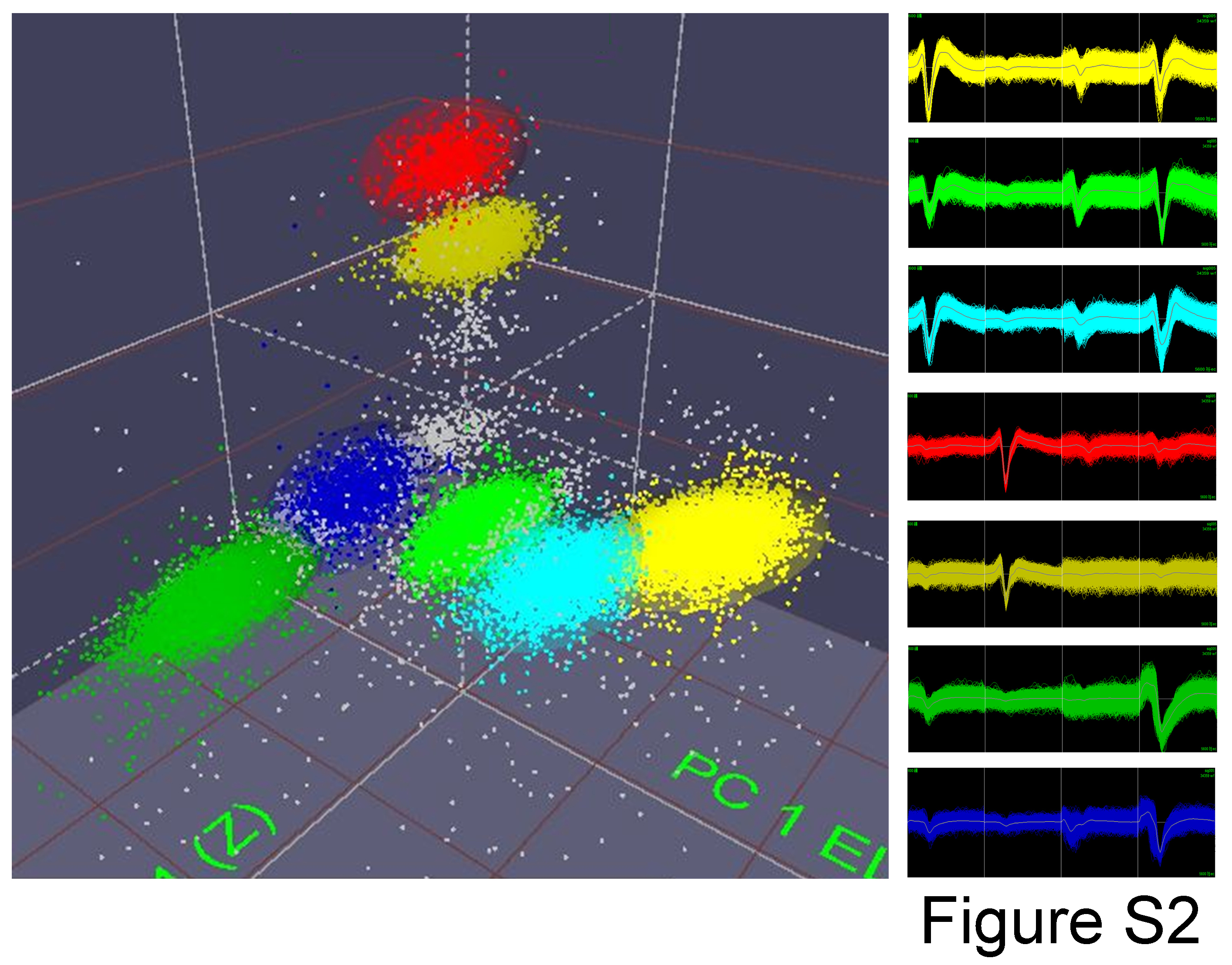

Supplement: Supplementary file 3 [file Image2.JPEG]
